# Topical Imiquimod Treatment of High-grade Cervical Intraepithelial Neoplasia (TOPIC-3): A Nonrandomized Multicenter Study

**DOI:** 10.1097/CJI.0000000000000414

**Published:** 2022-02-17

**Authors:** Natasja Hendriks, Margot M. Koeneman, Anna J.M. van de Sande, Charlotte G.J. Penders, Jurgen M.J. Piek, Loes F.S. Kooreman, Sander M.J. van Kuijk, Linde Hoosemans, Simone J.S. Sep, Peggy J. de Vos Van Steenwijk, Heleen J. van Beekhuizen, Brigitte F.M. Slangen, Hans W. Nijman, Roy F.P.M. Kruitwagen, Arnold-Jan Kruse

**Affiliations:** Departments of * Obstetrics and Gynecology; ∥ Pathology; ** Internal Medicine, Maastricht University Medical Center; † GROW—School for Oncology and Developmental Biology; ¶ Department of Methodology and Statistics; # CAPHRI—School for Care and Public health Research Institute, Maastricht University, Maastricht; § Department of Obstetrics and Gynecology, Catharina Hospital, Eindhoven; ‡ Department of Gynecological Oncology, Erasmus MC Cancer Institute, Rotterdam; †† Department of Obstetrics and Gynecology, University Medical Center Groningen, Groningen; ‡‡ Department of Obstetrics and Gynecology, Isala Clinics, Zwolle, The Netherlands

**Keywords:** CIN, HPV, imiquimod, LLETZ, immunotherapy

## Abstract

Topical imiquimod could be an alternative, noninvasive, treatment modality for high-grade cervical intraepithelial neoplasia (CIN). However, evidence is limited, and there are no studies that compared treatment effectiveness and side effects of topical imiquimod cream to standard large loop excision of the transformation zone (LLETZ) treatment. A multi-center, nonrandomized controlled trial was performed among women with a histologic diagnosis of CIN 2/3. Women were treated with either vaginal imiquimod (6.25 mg 3 times weekly for 8 to 16 wk) or LLETZ according to their own preference. Successful treatment was defined as the absence of high-grade dysplasia at the first follow-up interval after treatment (at 20 wk for the imiquimod group and at 26 wk for the LLETZ group). Secondary outcome measures were high-risk human papillomavirus (hrHPV) clearance, side effects, and predictive factors for successful imiquimod treatment. Imiquimod treatment was successful in 60% of women who completed imiquimod treatment and 95% of women treated with LLETZ. hrHPV clearance occurred in 69% and 67% in the imiquimod group and LLETZ group, respectively. This study provides further evidence on topical imiquimod cream as a feasible and safe treatment modality for high-grade CIN. Although the effectiveness is considerably lower than LLETZ treatment, imiquimod treatment could prevent initial surgical treatment in over 40% of women and should be offered to a selected population of women who wish to avoid (repeated) surgical treatment of high-grade CIN.

High-grade cervical intraepithelial neoplasia (CIN 2/3), or high-grade squamous intraepithelial lesions, is the premalignant condition of cervical cancer and is caused by cervical human papillomavirus (HPV) infection.^1^,^2^ Treatment is usually performed by large loop excision of the transformation zone (LLETZ). LLETZ is an effective intervention, but it is associated with an increased risk of premature birth in subsequent pregnancies.^3^ This risk increases with repeated interventions for recurrent lesions, which is required in up to 23% of women during long-term follow-up.^3^–^5^ In order to prevent unnecessary surgical treatment in women with a future pregnancy desire, nonsurgical treatment modalities could be explored.^6^

One such nonsurgical treatment modality could be immunotherapy with imiquimod. One previous study on imiquimod treatment of high-grade CIN has been conducted and showed promising results.^7^ Imiquimod is not labeled for the treatment of high-grade CIN. Disease regression or remission occurred in 73% of women in a randomized controlled trial in 59 women. In a patient preference study among women with high-grade CIN, we recently showed that women with a future pregnancy desire are generally willing to undergo imiquimod treatment.^8^ However, treating physicians feel an urgent need for further evidence on the effectiveness and safety of imiquimod treatment in high-grade CIN, before clinical implementation.^9^ The aim of the present study was therefore to provide additional evidence on effectiveness and side-effects of topical imiquimod cream in treatment of high-grade CIN, compared with standard LLETZ treatment, as well as the identification of predictive factors for successful imiquimod treatment.

## MATERIALS AND METHODS

### Study Design

The study was designed as a multicenter, nonrandomized clinical trial and was conducted in 3 hospitals in the Netherlands: Maastricht University Medical Center, Erasmus Medical Center Rotterdam and Catharina Hospital Eindhoven, between November 2016 and June 2018. Approval for this study was obtained from the Medical Ethics Committee of Maastricht University Medical Center (approval number: NL57849.068.16/METC162025, approval date September 5, 2016). ClinicalTrials.gov Identifier: NCT02917746. The manuscript was written according to the CONSORT guidelines.^10^

### Patient Population

Women aged 18 years and older, with a CIN 2 or CIN 3 diagnosis in cervical biopsies were assessed for eligibility. Exclusion criteria were previous histologically confirmed high-grade CIN, PAP4 cytology, concomitant vulvar and/or vaginal intraepithelial neoplasia, previous cervical malignancy, current malignant disease, immunodeficiency, pregnancy or lactation, legal incapability, and insufficient knowledge of the Dutch language. All women provided written informed consent before they were included in the study.

### Sample Size

The sample size calculation was based on an expected treatment effectiveness of 73% for imiquimod and 95% for LLETZ.^7^ With 80% power, an α of 5% and allowing for a withdrawal rate of 20%, 53 women would have to be recruited in each arm. This was rounded up to 60 women per arm, given the uncertainty of imiquimod effectiveness and withdrawal rate.

### Study Procedures

Women who were included in the study chose the treatment modality of their preference: either imiquimod treatment or LLETZ.

Imiquimod treatment consisted of 1 or 2 treatment periods of 8 weeks, with a colposcopy (including biopsies) in between to assess disease development during treatment. Imiquimod 5% cream was self-applied by use of a vaginal applicator in a dose of 6.25 mg (1/2 sachet), 3 times per week.^11^ Cream was administered in the evening and remainders rinsed in the shower the next morning. Tampon use was allowed from that moment in case of vaginal discharge (to prevent local side effects). Women recorded the occurrence and severity of side-effects on a daily basis, by use of a visual analog scale (VAS). In case of systemic drug-related side effects, women were first advised to use paracetamol and nonsteroidal anti-inflammatory drugs. In case of persistent systemic or local side effects, the frequency of imiquimod application was decreased to twice per week, subsequently to once per week and subsequently discontinued for 1 week if side effects persisted. Adequate contraception during the study was ensured. Study visits were scheduled at weeks 2, 6, and 14, in which treatment adherence and side-effects were assessed. Two weeks after the first treatment period (at 10 wk from baseline), a first colposcopy was performed. Biopsies were taken from the area(s) with original CIN 2/3 lesions and any other suspicious lesion. In case of histologic complete remission (no CIN), imiquimod treatment was stopped. In case of histologic regression to CIN 1 or stable disease, imiquimod treatment was continued for another 8 weeks. In case of progression, defined as either histologic progression in disease grade or an increase in visual lesions size with stable histologic disease grade, adequate surgical treatment was performed. All women, also those showing remission at 10 weeks, were scheduled to undergo cervical cytology for HPV genotyping and a colposcopy with biopsies at 20 weeks from baseline (2 wk after the second 8-wk treatment period), for evaluation of the primary outcome measure. After successful treatment, first follow-up cytology was performed after 6 months. A new colposcopy was planned in case of PAP3a2 or worse.

For women who opted for LLETZ treatment, this was performed within 4 weeks by monopolar loop electrode, under local anesthesia. Women recorded the occurrence and severity of side-effects on a daily basis, by use of a VAS, during 2 weeks. First follow-up was performed after 26 weeks (according to national protocol), by cytology and HPV genotyping. A repeat colposcopy with biopsies was planned in case of PAP3a2 or higher and additional treatment was performed in case of a persistent CIN 2/3 or worse.

All colposcopies and LLETZ procedures were performed or supervised by experienced gynecologists or gynecologic oncologists. Cytologic assessment was performed by trained cytology analysts, according to the Papanicolaou system.

### Laboratory Procedures

Histopathologic assessment of cervical biopsies was performed by a well experienced gynecologic pathologist according to national guidelines, according to the World Health Organization criteria, based on hematoxylin and eosin staining, with p16 and KI67 staining at disposal. HPV genotyping was performed using polymerase chain reaction enzyme immunoassays in either cytology or histology. DNA isolation of the samples was performed via Maxwell 16 (Promega). A PCR GP5+/6+ was run with HPV universal primers. HPV positivity was assessed via agarose gel electrophoresis. Positive HPV cases were subtyped using an Enzyme Immuno Assay with possible outcomes positivity for hrHPV16, HPV18, or cocktail hrHPV (31, 33, 35, 39, 45, 51, 52, 56, 58, 59, 66).

### Outcomes

The primary study outcome was successful treatment of high-grade CIN, defined as the absence of high-grade dysplasia after treatment, reflecting the clinical outcome that additional treatment of residual/recurrent disease is not necessary. For the imiquimod group, this was assessed by diagnostic biopsies at 20 weeks; for the LLETZ group this was assessed by cervical cytology after 26 weeks, followed by colposcopy with biopsies in case of PAP3a2 or worse.

Secondary outcomes were hrHPV status at primary outcome interval, the incidence and severity of treatment related side-effects and predictive factors for imiquimod treatment success.

### Statistical Analysis

Statistical analyses of the primary endpoint were performed according to both the intention-to-treat and per-protocol principle. The intention-to-treat analysis included all women that started the study, the per-protocol analysis included only those women who completed the full treatment scheme and were available for follow-up. Comparison between the 2 groups at baseline was performed with the independent samples *t* test. Comparison of treatment success, HPV clearance and side-effects between the two groups was performed using Pearson χ^2^ test. Patient characteristics that were potentially predictive of successful imiquimod treatment were identified using univariable logistic regression analysis. A *P*-value smaller than or equal to 0.05 was considered statistically significant. Statistical analyses were performed using IBM SPSS Statistics for windows (version 24.0; IBM Corp, Armonk, NY).

## RESULTS

**FIGURE 1:**
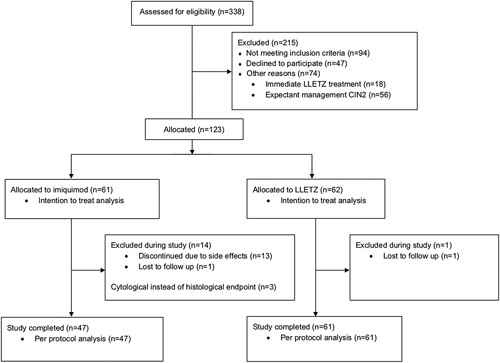
Study CONSORT diagram. CIN indicates cervical intraepithelial neoplasia; LLETZ, large loop excision of the transformation zone.

A total of 123 patients were included in this study. 61 women chose treatment with imiquimod and 62 women chose LLETZ treatment. The trial CONSORT diagram can be found in Figure 1. Baseline characteristics are presented in Table 1.

**TABLE 1:** Baseline Characteristics

Characteristic	Imiquimod (n=61)	LLETZ (n=62)	*P*
Age (y)			
Mean (SD)	33.3 (9.1)	35.2 (7.0)	0.21
BMI (kg/m^2^)*			
Mean (SD)	22.9 (3.5)	23.8 (4.2)	0.21
No. pregnancies			
Mean (SD)	0.7 (1.2)	1.6 (1.4)	<0.01
Nulliparity, n (%)			
Yes	45 (74)	20 (32)	<0.01
Previous pap abnormalities, n (%)			
Yes	9 (15)	6 (10)	0.39
Contraception, n (%)			
Oral contraception	25 (41)	23 (37)	0.58
Other hormonal contraception	12 (20)	20 (32)	
Other	1 (2)	1 (2)	
Smoking, n (%)			
No	36 (59)	36 (58)	0.99
Yes	22 (36)	22 (35)	
Quit within last 6 mo	3 (5)	4 (6)	
Sexual contacts, n (%)			
No sexual contact	9 (15)	13 (21)	0.61
Single sexual contact	43 (70)	43 (69)	
Multiple sexual contacts	8 (13)	6 (10)	
Unknown	1 (2)	0	
Histology at baseline, n (%)			
CIN 2	25 (41)	14 (23)	0.03
CIN 3	36 (59)	48 (77)	
HPV status, n (%)			
HPV 16/18	22 (36)	26 (42)	0.21
HPV 16/18 and other	3 (5)	4 (6)	
Other hrHPV	25 (41)	20 (32)	
HPV negative	10 (16)	12 (19)	
Unknown	1 (2)	0	
Time between initial biopsy and start treatment (d)			
Mean (SD)	30.4 (49.9)	26.6 (19.2)	0.58

* Missing for 1 patient in the imiquimod group.

### Treatment Adherence and Loss to Follow-up

In the imiquimod group, 13 women (21%) discontinued treatment, all due to side effects. One woman was unavailable for follow-up at 20 weeks. In women who continued imiquimod treatment, the mean number of applications was 20 (range 11–25 applications) during the first 8 treatment weeks and 17 (range 10–24 applications) in the second treatment period. In the LLETZ group, treatment adherence was 100%. One woman was not available for follow-up at 26 weeks.

### Treatment Effectiveness

#### Imiquimod Group

Outcomes at 10 and 20 weeks are summarized in Table 2. At 10 weeks, 4 women showed disease progression (increase in lesions size of a CIN 3 lesion) and underwent LLETZ. At the primary outcome interval at 20 weeks, 40 women underwent colposcopy with biopsies and three women underwent cytologic control instead of colposcopy (outside study protocol). These women were included in the per protocol analysis, in which PAP2 was regarded as successful treatment (n=2) and PAP3a2 (n=1) as unsuccessful treatment. Based on these results, the treatment success rate of imiquimod was 43% based on the intention-to-treat analysis and 60% based on the per protocol analysis (Table 3). All subsequent analyses include the 3 women for whom cytology was performed at 20 weeks.

**TABLE 2:** Outcomes at 10- and 20 Weeks Follow-up in the Imiquimod Group, for Women Who Continued Imiquimod Treatment Until at Least 10 Weeks

10 wk Colposcopy Outcomes (n=50*)	20 wk Follow-up: Mode and Number of Women		Outcomes Compared With Baseline (n=47)	
Remission†				
N=12	Colposcopy	N=10	Remission	N=5
			Regression to CIN 1	N=4
			Persistence	N=1
	Cytology	N=1	PAP 3a2	N=1
	Lost to FU	N=1		
Regression to CIN 1				
N=21	Colposcopy	N=18	Remission	N=6
			Regression to CIN 1	N=6
			Persistence	N=6
	Cytology	N=2	PAP 2	N=2
	Discontinued	N=1		
Regression to CIN 2				
N=5	Colposcopy	N=5	Remission	N=2
			Regression to CIN 1	N=2
			Persistence	N=1
Persistence CIN 2/3				
N=8	Colposcopy	N=7	Remission	N=1
			Regression to CIN 1	N=0
			Persistence	N=6
	Discontinued	N=1		
Progression				
N=4	NA, unsuccessful treatment	N=4	Unsuccessful treatment (persistence)	N=4

* Eleven women stopped due to side effects.

† Two women continued imiquimod treatment at their own request, one showed disease persistence of CIN 2 at 20 weeks, the other disease regression to CIN 1.

CIN indicates cervical intraepithelial neoplasia; FU, follow-up; NA, not applicable.

**TABLE 3:** Treatment Effectiveness

	Intention to Treat			Per Protocol		
	Imiquimod N=61	LLETZ N=62	*P*	Imiquimod N=47	LLETZ N=61	*P*
Successful treatment*	26 (43%)	58 (94%)	<0.01	28 (60%)	58 (95%)	<0.01

* Defined as the absence of high-grade dysplasia at the first follow-up interval after treatment (20 wk for imiquimod and 26 wk for LLETZ treatment), assessed by diagnostic biopsies for the imiquimod group and cervical cytology followed by colposcopy with biopsies on indication for the LLETZ group.

LLETZ indicates large loop excision of the transformation zone.

Follow-up cytology was first performed 6 months after completed treatment. Of the women who were successfully treated (n=28), 21 women (75%) had normal cytology and 4 women (14%) had PAP2 cytology. Three women (11%) had PAP3a1/2 cytology and underwent colposcopy. Histology revealed CIN 1 in 2 cases and CIN 3 in 1 case. Thus, disease recurrence at 6 months occurred in 4% (1/28) of women after successful imiquimod treatment.

#### LLETZ Group

At the primary outcome interval (26 wk), 3 women (5%) showed PAP 3a2 or higher and underwent a second colposcopy with biopsies. All 3 women were diagnosed with a residual CIN 2/3 lesion and underwent a second LLETZ. Based on these results, the treatment success rate of LLETZ was 94% based on the intention-to-treat analyses and 95% based on the per protocol analyses (Table 3).

### hrHPV Clearance

hrHPV clearance was evaluated for all women who were hrHPV positive at baseline and completed the study until the primary outcome interval. hrHPV clearance was comparable in the 2 treatment groups: 69% in women treated with imiquimod and 67% in women treated with LLETZ (Table 4). Of the women who were negative for hrHPV at baseline and underwent HPV testing at the primary outcome interval, all remained HPV negative.

**TABLE 4:** HPV Clearance After Completed Treatment

	Imiquimod Group (n=35)*	LLETZ Group (n=49)†	*P*
hrHPV clearance	24 (69%)	33 (67%)	0.91
HPV 16/18 clearance	9/15 (60%)	18/29 (62%)	0.89

* Included were women who were positive for hrHPV at baseline, completed imiquimod treatment and underwent colposcopy with biopsies at 20 weeks follow-up. Twenty-six women were excluded from the analysis: 13 women stopped treatment, 4 underwent LLETZ at 10 weeks, 1 was lost to follow-up, 3 HPV-tests failed, and 5 others were negative at baseline.

† Included were women who were positive for hrHPV at baseline. Thirteen women were excluded from the analysis: 12 were HPV negative at baseline and 1 was lost to follow-up.

HPV indicates human papillomavirus; hrHPV, high-risk human papillomavirus; LLETZ, large loop excision of the transformation zone.

hrHPV clearance in relation to treatment outcome was evaluated for the imiquimod group only. hrHPV clearance occurred in 20 of 23 (87%) women with successful treatment, compared with 4 of 12 women (33%, *P*<0.01) with unsuccessful treatment.

### Side Effects

In the imiquimod group, 13 women (21%) discontinued treatment due to side effects. Treatment discontinuation due to side-effect differed according to inclusion hospital, with rates of 9% (1/11), 13% (5/38), and 58% (7/12). For those who continued treatment, outcomes regarding side effects are presented in Table 5. All women experienced side effects, most commonly headache, fatigue, myalgia, and vulvar pruritus/pain. Thirty women (69%) reported 1 or more severe side effects (VAS ≥8).

**TABLE 5:** Side-effects of Imiquimod Treatment, Reported First 8 Weeks*

	Side Effect, Overall		Side Effect, VAS ≥8	
N=42	No. Patients (%)	Mean Duration (d)	No. Patients (%)	Mean Duration (d)
Headache	39 (93)	13.9	22 (52)	3.3
Fever	29 (69)	4.5	13 (31)	2.4
Fatigue	36 (86)	11.4	16 (38)	6.4
Myalgia	34 (81)	8.9	11 (26)	4.5
Vaginal discharge	29 (69)	10.4	6 (13)	3.5
Vaginal blood loss	27 (64)	6.0	5 (12)	5.2
Vulvar pruritus/pain	34 (81)	9.5	16 (38)	3.1
Vulvar redness	18 (43)	2.4	6 (14)	4.7

* Outcomes were available for 42 of 50 women (84%) who completed the first 8 weeks of the treatment protocol, but for only 14 women in the second 8 weeks. Therefore, only side effects reported in the first 8 weeks were analyzed and presented.

Outcomes regarding side-effects in the LLETZ group are presented in Table 6. All women experienced side effects, most commonly vaginal discharge and vaginal blood loss. Thirteen women (29%) experienced 1 or more severe side effects (VAS ≥8), significantly less than in the imiquimod group (*P*<0.01).

**TABLE 6:** Side-effects of LLETZ Treatment, Reported During 2 Weeks

	Side Effect, Overall		Side Effect, VAS ≥8	
N=45*	No. Patients (%)	Mean Duration (d)	No. Patients (%)	Mean Duration (d)
Abdominal pain	35 (78)	4.8	4 (9)	1.5
Vaginal discharge	45 (100)	8.9	14 (31)	3.8
Vaginal blood loss	45 (100)	9.0	23 (51)	2.2
Vaginal pruritis/pain	7 (16)	4.6	4 (9)	3.0
Fever	2 (5)	1.0	0	0.0
Hemorrhage with intervention	3 (5†)	—	—	—

* Outcomes were available for 45 of 62 women (73%).

† As a percentage of all women who underwent LLETZ.

LLETZ indicates large loop excision of the transformation zone; VAS, visual analog scale.

### Subgroup Analyses of Responders Versus Nonresponders in Imiquimod Group

None of the potential predictors were identified as a statistically significant predictive factor for imiquimod treatment outcome, although the OR’s for nulliparity, previous abnormal cytology and smoking indicate clinically meaningful associations (Table 7).

**TABLE 7:** Univariable Logistic Regression Analysis of Potential Predictors for Successful Imiquimod Treatment (N=47)

	OR (95% CI)	*P*
Age (y)	1.07 (0.97–1.17)	0.25
Nulliparity	3.57 (0.77–16.54)	0.10
Single sexual partner	1.00 (0.27–3.76)	1.00
Previous abnormal cytology	0.16 (0.02–1.40)	0.10
CIN II at diagnosis	0.91 (0.29–2.91)	0.88
Smoking (recently quit=no)	0.49 (0.15–1.63)	0.24
Smoking (recently quit=yes)	0.33 (0.10–1.09)	0.07
No. imiquimod applications	1.00 (1.00–1.00)	0.81
HPV 16/18 infection at baseline	1.00 (0.98–1.00)	0.54

CI indicates confidence interval; CIN, cervical intraepithelial neoplasia; HPV, human papillomavirus; OR, odds ratio.

## DISCUSSION

This study shows that topical imiquimod was successful in 60% of patients who completed treatment in the per protocol analysis, and 43% in the intention to treat analysis. After successful imiquimod treatment, hrHPV clearance was comparable to LLETZ and disease recurrence at 6 months occurred in only 4% of women. Although treatment success is lower for imiquimod than for LLETZ, our findings indicate that imiquimod treatment can avoid LLETZ in a significant number of women with CIN 2/3. As such, imiquimod could be a feasible treatment alternative to LLETZ, for a selected population of women who want to avoid the surgical treatment approach.

This study has several strengths. It is the largest study so far evaluating imiquimod treatment of high-grade CIN and the first study comparing imiquimod treatment to LLETZ. After we learned that women had a clear preference for either of the 2 treatment modalities, we deliberately chose for an open-label design, which reflects a “real life” scenario.^8^,^12^ This design inevitably leads to baseline differences between the treatment groups: in this study differences in parity and CIN grade. These factors may influence treatment outcomes, but do represent a “real life scenario” comparable to clinical practice.

The study also has several limitations. First, 3 women in the imiquimod group only had cytology available at the primary endpoint, but were included in the analyses nevertheless. Although their PAP results strongly suggested either treatment success or treatment failure, their results would have been more reliable had histology been available. Second, we cannot rule out a positive effect on disease regression of the biopsies taken at the 10-week colposcopy, due to actual removal of dysplastic tissue and activation of the immune system to induce spontaneous regression. However, we justify this procedure in the context of safety. A final limitation of the study is the difference in outcome assessment and timing between the 2 groups (histology at 20 wk for the imiquimod group and cytology followed by colposcopy and biopsies on indication at 26 wk for the LLETZ group). The different procedures were chosen with consideration of safety in the imiquimod group, prevention of unnecessary/invasive investigations for women treated with LLETZ and adherence to guidelines concerning follow-up after LLETZ. It is unknown whether these differences have influenced study outcomes. However, the selected outcome measures and procedures reflect the measures and procedures as they will be used in clinical application.

Imiquimod effectiveness in this study is lower than reported by Grimm et al,^7^ despite similar treatment regimens, applied doses, and baseline histology. A potential explanation could be the different mode of administration (applicator vs. suppository), but the difference in effectiveness may also represent a natural distribution in different populations. The effectiveness of imiquimod is also significantly lower than LLETZ effectiveness. The definition of clinically acceptable effectivity thresholds is subject to debate. Thresholds of 45%–60% have been postulated by clinicians, but the more important is what patients themselves desire.^13^ A previous study by the authors showed that, whereas a general population of women required a treatment effectiveness of 95% to prefer imiquimod over LLETZ, women with a future pregnancy desire would accept a lower treatment success rate of 72%.^8^ This indicates that imiquimod may not qualify as a treatment alternative for all women, but that it could be a good option for women with a future pregnancy desire. In order to meet the desired treatment effectiveness threshold—which is higher than in our study—predictive factors for treatment success could be applied to identify women in whom a treatment success higher than 72% can be expected. Our study has not been able to identify such factors so far. Future studies, for example on biomarkers, could be performed to this aim.

All women, whether they were treated with imiquimod or underwent LLETZ, reported side effects, but severe side effects occurred more frequently in women treated with imiquimod. A selection of these side effects has been reported by the authors in a previous publication.^14^ Although the total number of women with side effects in our study is similar to the study by Grimm et al, they reported less treatment discontinuation (3%) and less severe (grade 2) side effects.^7^,^14^ There may be several explanations for this. First, treatment discontinuation differed significantly between the inclusion hospitals in our study. This may reflect differences in counselling or women’s expectations and carrying capacity, but also a difference in management of side-effects. Indeed, a lower treatment discontinuation was seen in the primary inclusion center, where counselling and management was provided by a specialized nurse practitioner, who was easily accessible to the women in the study. Other explanations could be related to the treatment regimen in general: we did not apply a step-up regimen or dose reduction, such as Grimm et al.^7^ A final explanation may be the difference in imiquimod administration: our study used cream in applicators instead of suppositories. Overall, it can be concluded that imiquimod treatment is associated with significant side effects, which is an important issue in the counselling of women for this treatment. Moreover, adequate management of side-effects and support during treatment is important to minimize treatment discontinuation.

The hrHPV clearance rate in our study is comparable to the study by Grimm et al^7^ and confirms that imiquimod is efficacious in clearing HPV infection. This is important with regard to the fact that prior studies have shown that HPV clearance is predictive of maintaining disease resolution of high-grade CIN.^15^ In line with Grimm and colleagues, we found no statistically significant differences in hrHPV types in women with successful or unsuccessful imiquimod treatment, indicating that there are no differences in effectiveness of imiquimod in treating a specific type of HPV.

Although treatment success of imiquimod for high-grade CIN is considerably lower than treatment success of LLETZ, imiquimod treatment could prevent initial surgical treatment in over 40% of women. Imiquimod treatment leads to adequate HPV clearance and has a low recurrence rate at 6 months posttreatment. As such, topical imiquimod could be a feasible nonsurgical treatment alternative for a selected population of women who want to avoid (repeated) surgical treatment, such as women with a future pregnancy desire. Careful counselling on side-effects and their adequate management is important. To enable selection of women with a high likelihood of successful imiquimod treatment, studies are needed that identify predictive (bio)markers for imiquimod treatment outcome. Following on the outcomes of this study, this will lead to optimal opportunities for a personalized treatment strategy for high-grade CIN.
